# Clove Oil-Based Nanoemulsion Containing Amphotericin B as a Therapeutic Approach to Combat Fungal Infections

**DOI:** 10.3390/pharmaceutics17070925

**Published:** 2025-07-17

**Authors:** Marcel Lucas de Almeida, Ana Paula dos Santos Matos, Veronica da Silva Cardoso, Tatielle do Nascimento, Ralph Santos-Oliveira, Leandro Machado Rocha, Francisco Paiva Machado, Franklin Chimaobi Kenechukwu, Alane Beatriz Vermelho, Eduardo Ricci-Júnior

**Affiliations:** 1 Laboratório de Desenvolvimento Galênico, Farmácia Universitária, Universidade Federal do Rio de Janeiro, UFRJ, Rio de Janeiro 21941-901, Brazil; marcellucalmeida@gmail.com (M.L.d.A.); tatiellenascimento94@gmail.com (T.d.N.); 2 Institute of Drug Technology-Farmanguinhos, Oswaldo Cruz Foundation (Fiocruz), Rio de Janeiro 21040-900, Brazil; anapaulasmatos@gmail.com; 3 Laboratório de Biotecnologia BIOINOVAR (Unidade de Biocatálise, Bioprodutos e Bioenergia), Universidade Federal do Rio de Janeiro, UFRJ, Rio de Janeiro 21941-901, Brazil; verocardoso@micro.ufrj.br (V.d.S.C.); abvermelho@micro.ufrj.br (A.B.V.); 4 Laboratory of Nanoradiopharmacy and Synthesis of Novel Radiopharmaceuticals, Nuclear Engineering Institute, Rio de Janeiro 21941-598, Brazil; presidenciaradiofarmacia@gmail.com; 5 Laboratory of Nanoradiopharmacy and Radiopharmaceuticals, Zona Oeste State University, Rio de Janeiro 20550-013, Brazil; 6 Laboratório de Tecnologia de Produtos Naturais, Universidade Federal Fluminense (UFF), Niterói, Rio de Janeiro 24210-201, Brazil; lean.machado@gmail.com (L.M.R.); fmachado@id.uff.br (F.P.M.); 7 Drug Delivery and Nanomedicines Research Group, Department of Pharmaceutics, Faculty of Pharmaceutical Sciences, University of Nigeria, Enugu 410105, Nigeria; frankline.kenechukwu@unn.edu.ng

**Keywords:** nanoemulsion, clove oil, amphotericin B, in vitro release studies, candidiasis, sporotrichosis

## Abstract

**Background/Objectives:** Candidiasis, primarily caused by *Candida albicans*, and sporotrichosis, mainly caused by *Sporothrix schenckii*, are skin fungal infections that pose serious threats to global health. The *Candida auris* is a great concern in immunocompromised individuals, and while *Sporothrix brasiliensis* cause sporotrichosis, an infection commonly found in cats, this disease can be transmitted to humans through scratches or bites. Existing treatments for these fungal infections often cause problems related to resistance and significant side effects. Consequently, development of alternative therapeutic approaches such as nanotechnology-based topical lipid-based formulations is interesting. Thus, the objectives of this study were to prepare clove oil (CO)-in-water nanoemulsions (NEs) containing amphotericin B (AmB) and characterize them with respect to stability, release profile, and in vitro cytotoxic activity against *Candida* and *Sporothrix* strains. As a future alternative for the treatment of fungal skin diseases. **Methods:** Chemical analysis of clove oil was obtained by GC-MS. The NEs were produced using an ultrasound (sonicator) method with varying proportions of CO, Pluronic^®^ F-127, and AmB. The NEs were characterized by droplet size, morphology, stability and in vitro release profile. The antifungal and cytotoxic activity against *C. albicans*, *C. auris*, *S. schenckii*, and *S. brasiliensis* were ascertained employing agar diffusion and colorimetric MTT assay methods. A checkerboard assay was carried out using clove oil and amphotericin B against *C. auris*. **Results:** Eugenol was the major compound identified in CO at a concentration of 80.09%. AmB-loaded NEs exhibited particle sizes smaller than 50 nm and a polydispersity index below 0.25. The optimal Ne (NEMLB-05) remained stable after 150 days of storage at 4 °C. It exhibited rapid release within the first 24 h, followed by a slow and controlled release up to 96 h. NEMLB-05 more effectively inhibited *C. auris* compared to free AmB and also demonstrated greater activity against *C. albicans*, *S. schenckii*, and *S. brasiliensis*. Clove oil and amphotericin B presented synergism inhibiting the growth of *C. auris*. **Conclusions:** The selected CO-in-water NEs containing AmB demonstrated promising potential as a topical therapeutic alternative for treating fungal infections.

## 1. Introduction

The skin is the primary route for disease treatment; however, its natural barriers, the cellular layers of the epidermis and dermis, pose significant challenges to drug penetration into infected cells. Only a limited number of drugs with suitable physicochemical properties can cross these barriers, making skin infections particularly challenging to eradicate [1]. *Candida albicans* is a dimorphic fungus that naturally lives as a commensal on barrier surfaces like the skin, though it can also become pathogenic. In individuals with compromised immune function, *C. albicans* can enter the bloodstream, leading to disseminated candidiasis [2,3].

The development of invasive candidiasis typically requires an increase in fungal load and changes in the skin or mucosal surfaces [3]. In contrast, *C. auris* effectively colonizes human skin and can persist on non-living surfaces, this condition facilitates its rapid spread in healthcare settings contributing to outbreaks of infection. Patients at particularly high risk include those in intensive care units and individuals with immune impairments due to previous surgery, trauma, chronic kidney disease, or diabetes [4,5].

Sporotrichosis is a subcutaneous mycosis caused by thermally dimorphic fungi of the genus *Sporothrix*. *Sporothrix schenckii* is a fungus with great potential to cause the Sporotrichosis, which is recognized as the primary global cause of human mycosis and is typically found in warm, tropical, and subtropical climates [6]. Sporotrichosis usually develops following contact with environmental spores, particularly through the skin. It is primarily transmitted via minor skin injuries that occur during the handling of soil or decomposing vegetation contaminated with the fungus. Recently, *Sporothrix brasiliensis* has been identified in urban cats, in countries like Brazil. These animals are susceptible to infection, and there have been reports of transmission to humans through bites and scratches, highlighting the importance of treating infected animals to reduce human transmission [6,7].

Fungal cell membranes predominantly contain ergosterol, making them a prime target for antifungal strategies to inhibit its synthesis, such as with azoles. The therapeutic arsenal against candidiasis includes three classes of antifungal agents: polyenes (e.g., amphotericin B–AmB), azoles–imidazoles and triazoles (e.g., clotrimazole, miconazole, fluconazole and posaconazole), and echinocandins—with semi-synthetic derivatives like caspofungin, micafungin, and anidulafungin. Polyenes can cause severe nephrotoxicity, because they act non-selectively on cholesterol in mammalian membranes. Meanwhile, echinocandins require intravenous administration and are costly, limiting their widespread use. Furthermore, azoles have fungistatic effects on *Candida* and *Sporothrix* species, but, indiscriminate use can lead to the emergence of azole resistance in other strains such as *C. auris* [8,9].

The standard treatment for sporotrichosis usually involves itraconazole, either alone or in combination with potassium iodide. Unfortunately, this therapy often causes side effects such as loss of appetite, depression, and hepatotoxicity. The long treatment duration—often spanning several months—can complicate recovery, particularly in cats. Consequently, there is an urgent need for new and alternative regimens and treatments that can be applied directly to the skin. Other treatment options, such as AmB, terbinafine, posaconazole, local heat therapy, and cryosurgery, have been described, although their efficacy varies considerably [7,10].

Therefore, given the challenges with current treatments for candidiasis and sporotrichosis, exploring alternative therapeutic options is crucial. Essential oils, with well-known antimicrobial properties, represent a promising alternative, especially utilizing technologies like nanoparticles.

One potential approach for developing new therapeutic alternatives is nanoemulsions (NEs). NEs are nanometric scale systems (<200 nm) made up of two immiscible liquids, typically oil and water. They consist of tiny droplets of one immiscible substance, such as oil, dispersed in the other. The addition of surfactants like Pluronic^®^ F-127 is essential for forming these droplets [11,12].

Previous reports have shown that clove oil (CO) NEs were typically formed by combining CO with surfactants using high-shear mixing techniques, such as high-pressure homogenization and sonication, reducing the oil droplets to nanometric scales [13,14]. The smaller particle size of the developed formulations provided a larger contact surface area, which enhanced drug dispersion and skin absorption. Interestingly, CO is inherently known for its various therapeutic activities, such as antiseptic and antimicrobial properties [15,16], and its main constituent, eugenol (phenylpropanoid), is also believed to have these properties [17,18].

Advantageously, natural products such as CO are considered safer and more cost effective for exploration in synergy with other antifungal drugs, such as AmB, which has poor water solubility and struggles to cross epithelial barriers [19,20]. Therefore, the development of nano-sized carriers that can enhance the bioavailability of AmB in the body is of great interest to public health [21]. Pluronic^®^ F-127 is a biocompatible polymeric surfactant, and is extensively used in the preparation of nanoemulsions. Our research group has used this surfactant for the preparation of nanoemulsions [13].

The aim of this study was to develop clove oil-in-water NEs containing AmB, as a therapeutic alternative for the treatment of fungal infections that affect human skin. The NEs were prepared via ultrasound technique, characterized for stability, release profile, and in vitro cytotoxic activity against *Candida* and *Sporothrix* strains.

## 2. Materials and Methods

### 2.1. Materials

The following materials were used in this study: Clove oil (*Syzygium aromaticum* (L.) Merr. & L.M.Perry, Ferquima, São Paulo, Brazil); Pluronic^®^ F-127 (Sigma-Aldrich, São Paulo, Brazil); AmB (Xellia Pharmaceuticals, Copenhagen, Denmark); dimethyl sulfoxide (DMSO; Tedia, Rio de Janeiro, Brazil); dichloromethane (≥99.9%, Sigma-Aldrich, St. Louis, MO, USA), acetonitrile (Tedia, Rio de Janeiro, Brazil), methanol (Tedia, Rio de Janeiro, Brazil), sodium lauryl sulfate (Spectrum, Rio de Janeiro, Brazil); potassium chloride (KCl; Infinity Pharma, Rio de Janeiro, Brazil); anhydrous disodium phosphate (Na_2_HPO_4_; Vetec, Rio de Janeiro, Brazil); 3-(4,5-dimethyl-2-thiazolyl)-2,5-diphenyl-2H-tetrazolium bromide (MTT; Sigma-Aldrich, São Paulo, Brazil); and Dulbecco’s modified Eagle medium (DMEM; Sigma-Aldrich, São Paulo, Brazil); Resazurin (Sigma-Aldrich, São Paulo, Brazil). All the reagents used in this study were analytical grade.

### 2.2. Methods

#### 2.2.1. Chemical Characterization of *Syzygium aromaticum* Essential Oil

The components of the essential oil of *Syzygium aromaticum* (L.) Merr. & L.M.Perry were analyzed by gas chromatography coupled with mass spectrometry (GC-MS QP2010, Shimadzu, Kyoto, Japan) and quantified in a gas chromatograph (GC-2014, Shimadzu, Kyoto, Japan) coupled to a flame ionization detector (FID). The essential oil (diluted to 1000 ppm in dichloromethane, ≥99.9%, Sigma-Aldrich, St. Louis, MO, USA) was injected at a volume of 1 µL, using an injector set to 260 °C with a 1:10 split ratio. The chromatographic conditions were as follows: helium (White Martins Corp., Rio de Janeiro, Brazil) was used as the carrier gas with a flow rate of 1 mL/min and RTX-5MS capillary column (Restek Corporation, Bellefonte, PA, USA; 36 id. 0.25 mm, 30 m length, 0.25 µm film thickness). The initial oven temperature is 60 °C, with a 3 °C/min increase until 290 °C. Mass spectrometry (MS) was performed at 70 eV with a scan rate of 1 scan/s. GC-FID chromatographic conditions were similar to the MS, except for the injection in an RTX-5 column (Restek corporation, id. 0.25 mm, 30 m length, 0.25 µm film thickness, Bellefonte, PA, USA) and 290 °C FID temperature. The arithmetic index (AI) was calculated by interpolating the retention times of the saturated alkanes standard (C7-C40, Sigma-Aldrich, St. Louis, MO, USA) analyzed under the same chromatographic conditions. The essential oil substances were identified by comparing their retention indices and mass spectra. The relative abundance of the constituents was obtained using the FID peak normalization method.

#### 2.2.2. First Experimental Design

Based on the results of previous experiments [13], the design of central composite (CCD) was applied as the experimental design. The study was carried out with two replicates at the central point. The aim was to determine the optimal ratio of CO and Pluronic^®^ F-127 for encapsulating AmB. The analysis was conducted using Statistica 10 software (StatSoft). Two independent variables were considered in the experimental design: CO and Pluronic^®^ F-127 (Table 1).

The 10 formulations suggested in the experimental design were prepared by varying the concentrations of CO and Pluronic^®^ F-127 (Table 2).

The NE preparation consists of dissolving the surfactant (Pluronic^®^ F-127) in distilled water and placing it in a tube. Thereafter, CO was added to the tube and sonicated (frequency 20 kHz) using a SONICS Vibra Cell VCX130 (Newtown, CT, USA) for 10 min in pulse mode (15 s on + 5 s off) with 100% amplitude with the tube immersed in an ice bath to prevent it from heating up. The total volume of each formulation was 10 mL.

#### 2.2.3. Second Experimental Design and Formulation Selection

Based on the results from the first experimental design, a second CCD experimental design was conducted, varying the concentrations of CO and AmB, while keeping the Pluronic^®^ F-127 concentration at 10% (Table 3).

The 10 formulations suggested in the second experimental design were developed by varying the concentrations of CO and AmB (Table 4).

**Figure 1:**
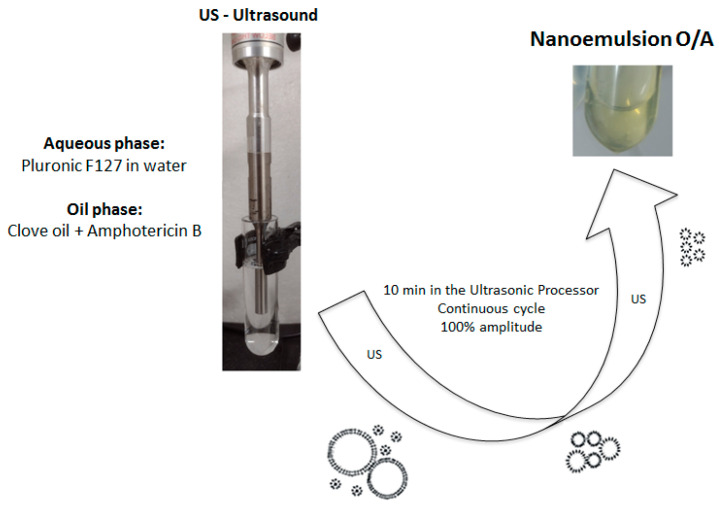
Key steps in the preparation of nanoemulsions containing amphotericin B.

The steps for developing the NEs were as follows: First, Pluronic^®^ F-127 (10%) was dissolved in distilled water and thereafter refrigerated. Then, AmB (0.021–0.045%) was dissolved in DMSO (ratio of 1 mg of AmB to 60 µL of DMSO) followed by addition of CO. The ultrasound was turned on and the drug solution was added drop by drop to the tube (25 mL) containing the surfactant solution for five minutes. After adding the drug solution, the ultrasound remained on for another five minutes, totaling 10 min for the ultrasonic process. The process was carried out for 10 min in an ice bath, in pulsation mode (15 s on + 5 s off) with 100% amplitude. The total volume of each formulation was 10 mL. Figure 1 shows the steps in the preparation of the formulations using high-energy ultrasound.

The two most promising NEs were selected for the stability, release, and antimicrobial activity analyses. The first formulation, NEMLB-05, contained 10% Pluronic^®^ F-127 (surfactant), 7.5% CO, and 0.035% AmB. The second formulation, NEMLB-06, contained 10% Pluronic^®^ F-127 (surfactant), 5% CO, and 0.025% AmB. These concentrations were chosen based on the results obtained in the first (Table 1) and second experimental designs (Table 3); they demonstrated excellent particle size and a low polydispersity index (PDI).

#### 2.2.4. Physicochemical Characterization of Clove Oil Nanoemulsions

The particle size (diameter in nm) and PDI of the NEs were determined using the dynamic light scattering (DLS) method with a Zetasizer Nano model S90 (Malvern Panalytical, Doncaster, UK). The NEs were diluted in distilled water (1:50) and measured in triplicate at room temperature (±26 °C). The pH of the formulations was assessed using a pH meter (PHS3BW, BEL Engineering, Milan, Italy).

The characterization of AmB and the content of the NEMLB-05 and NEMLB-06 formulations were determined using spectrophotometry in the visible and ultraviolet regions (UV–Vis spectrophotometer model V-630, Jasco, Tokyo, Japan). In this experiment, 1 mg of AmB was weighed and dissolved in DMSO to a final volume of 10 mL (100 µg/mL) in a volumetric flask. After solubilization, the solution was diluted 100× with methanol, resulting in a concentration of 1 µg/mL for analysis. A 2.5 mL sample was obtained from the solution and placed in the appropriate cuvette to identify the wavelength at which AmB showed the highest absorbance. It was also confirmed that CO and phosphate buffer with sodium lauryl sulfate did not have absorbance at the same wavelength as AmB. Therefore, the method showed selectivity for quantifying AmB in the formulations.

An analytical curve was plotted to calculate the AmB content. First, 1 mg of AmB was dissolved in 10 mL of DMSO to prepare a 100 µg/mL stock solution. Successive dilutions were made, reaching a minimum concentration of 0.25 µg/mL. Absorbance readings were obtained at a wavelength of 405 nm. Microsoft Excel 2016 was used to construct the analytical curve and analyze the content of the formulations.

NEMLB-05 and NEMLB-06 were subjected to a long-term stability analysis. The selected formulations were stored under two different temperature conditions: 28 ± 2 °C (room temperature) and 4 ± 2 °C (refrigerated). The color, odor, particle size, and PDI were analyzed on days 7, 18, 53, 83, 111, and 150 for NEMLB-06, and on days 8, 15, 36, 64, 100, and 150 for NEMLB-05.

A Tecnai G2 Spirit Bio-twin (FEI Company, Hillsboro, OR, USA) transmission electron microscope (TEM) was used to obtain the images of the NEs. The NEMLB-06 formulation was diluted with 10 µL of the formulation added to 90 µL of water (1:9), without the use of contrast. Next, 5 µL of the diluted NE was placed onto a 300-mesh copper grid suitable for the microscope. The grid was allowed to dry for 2 h before being placed in the microscope for imaging and analysis.

#### 2.2.5. In Vitro Release Analysis

The release analysis was performed with NEMLB-05, NEMLB-06, and free AmB in DMSO. The analytical curve for AmB was constructed along with the in vitro release experiment. To develop the analytical curve, 1 mg of AmB was dissolved in 10 mL of DMSO (100 µg/mL) and used as the stock solution. This solution was then subjected to several dilutions to obtain the required AmB concentrations for the analytical curve (0.25–7 µg/mL). The curve was generated using spectrophotometric analysis in the UV–visible range for the concentrations prepared in µg/mL, with the results being plotted in Microsoft Excel 2016 for the construction of the analytical curve. The method’s selectivity was assessed by spectrophotometric analysis of (i) AmB diluted in DMSO, (ii) AmB diluted in the receptor medium used for the in vitro release analysis, (iii) AmB encapsulated in NEs, (iv) and NEs (without the drug) diluted in the receptor medium.

**Figure 2:**
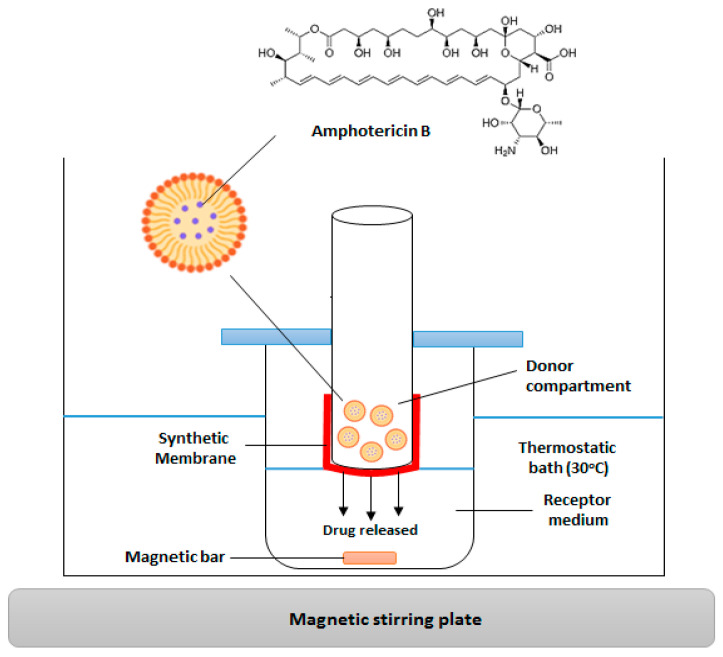
Release of amphotericin B from the nanoemulsions.

Four beakers were prepared for the release analyses, each containing 5 mL of the receptor medium consisting of phosphate buffer with 0.5% sodium lauryl sulfate at pH 7.4. Sodium lauryl sulfate was used to improve the solubility of AmB in the receptor medium. Cellulose membranes (D9652-100FT, Sigma-Aldrich, São Paulo, Brazil) were used for the release process. The membranes were hydrated in water for 3 h before being attached to a plastic tube with string, ensuring the membrane surface was in contact with the receptor medium. An amount of 100 µL (25 µg of AmB) of the formulation was placed inside the tube with the membrane. The beakers were placed in a glass box with water in a water bath at 30 ± 2 °C, on an IKA^®^ RT 15 (Staufen, Germany) shaker, where they were kept in constant agitation (300 rpm) for 96 h (Figure 2).

At pre-established time intervals (1, 3, 24, 48, 72, and 96 h), 2.5 mL aliquots were withdrawn from the receptor medium and analyzed using a spectrophotometer (V-630, Jasco, Tokyo, Japan) at the maximum absorption wavelength of AmB (λ = 412 nm). After each measurement, the aliquots were returned to the receptor medium to continue the experiment for up to 96 h. The results were recorded in a spreadsheet, and the necessary calculations were performed to determine the amount of AmB released at each time point in relation to the total AmB.

The release mechanism of AmB encapsulated in NEs was elucidated by analyzing the release kinetics. The data obtained from in vitro release analyses of AmB contained in NEs were fitted to various kinetic models: Zero Order, First Order, Higuchi, Korsmeyer–Peppas, Hixson–Crowell, Hopfenburg, Baker–Lonsdale, Peppas–Salin, Weibull, Logistic, Gompertz, and Probit using the DDSolver software version 1.0, software developed as a complementary program for Microsoft Excel. The release data were fitted to the kinetic models and linearization; the adjusted determination coefficient (R^2^) values and the model selection criterion (MSC) obtained for each model were compared [22]. The model providing the highest adjusted R^2^—consequently, the highest MSC value—was considered the kinetic model that best fits the AmB release results from NEs [23,24,25].

#### 2.2.6. In Vitro Biological Activity

##### Antifungal Activity by Agar Disc Diffusion of Nanoemulsions

The cells originated from the American Type Culture Collection (ATCC), Manassas, VA, USA. The cells were cultured and stored at the Bioinovar laboratory, Rio de Janeiro, Brazil. The following strains were used in the experiment: *C. albicans* (ATCC SC5314), *C. auris* (ATCC MYA-5002), *S. schenkii* (S15: ATCC 15383), and *S. brasiliensis* (S5: ATCC 5110). Initially, the cells (10^6^ cells per ml) were inoculated into Sabouraud dextrose agar (SDA) medium (for *Candida* growth) or BHI (brain heart infusion) medium (for *Sporothrix* growth). The cells (100 uL) were spread in all directions over the surface of the medium (25 mL) on a Petri dish (90 × 15 mm^2^). Subsequently, 10 μL of the NEMLB-05 and NEMLB-06 formulations containing AmB and the two formulations without AmB (NESAF-06 control for NEMLB-06; NESAF-09 control for NEMLB-05) were placed onto sterile paper discs (Laborclin Paraná, Pinhais, Brazil) positioned on the Petri dish. The plates were incubated for 24 h at 37 °C, and all tests were conducted in triplicate. After the incubation period, the inhibition zone (in cm) of fungal growth was measured planimetrically. Fluconazole (2.5 mg/mL) was used as the standard antifungal in the experiments.

##### Antifungal Activity by Microdilution of Nanoemulsions

The best formulations containing AmB (NEMLB-05) were tested for antimicrobial activity, and the equivalent formulation without AmB was also tested (NESAF-09). The experiment utilized the strains *C. albicans* (ATCC SC5314), *C. auris* (ATCC MYA-5002), *S. schenkii* (ATCC 15383), and *S. brasiliensis* (ATCC 5110) in 96-well plates using the colorimetric MTT assay. For the test, 100 µL of the medium was added to each well, followed by 100 μL of the filtered formulations (using a 0.22 µm filter) added to the first well. A serial dilution was performed, obtaining various concentrations of the samples, ranging from 75 to 0.391 mg/mL for CO and 0.35 to 0.00195 mg/mL for AmB. Next, 100 µL of a 10^6^ cells/mL yeast suspension was added to each well of the microplate (except for the first row, which served as the positive control), and the plates were incubated at 37 °C for 24 h for *Candida* and 48 h for *Sporothrix*. The experiment was performed in triplicate for *Candida* and with nine replicates for *Sporothrix*. After the incubation period, 20 µL of a 0.5% aqueous MTT solution was added to each well, and the microplates were incubated at 37 °C for an additional 3 h. After this period, the plates were centrifuged for 10 min at 300 G, and the supernatant from each well was discarded. Then, 200 µL of DMSO was added to each well to dissolve the formed crystals [26]. The concentration of the sample that reduces fungal proliferation by 50% (IC_50_) was determined by measuring the absorbance of the wells at 490/570 nm using the Varioskan Lux model VLBL00D0 (Thermo Scientific, Waltham, MA, USA). Microsoft Excel 2016 was used for regression analysis.

##### Checkerboard Assay

The study used a combination of two active ingredients, clove oil and amphotericin B, to determine whether there is synergy between them. The methodology was performed through a serial microdilution in a 96-well plate of clove oil horizontally and AmB vertically, obtaining a combination of different concentrations, in triplicate. In addition, a serial dilution of each of them alone was performed on the same plate [27]. *C. auris* cells were used for the study. In each well, 100 μL of the culture medium, 100 μL of each active ingredient, and 100 μL of the fungus were added. The microplates were incubated at 28 °C, and after a period of 2 days, resazurin was added (25 μL). After 2 h, the interactions between the clove oil and AmB were visually read and analyzed, based on their respective minimum inhibitory concentration (MIC) indices.

The synergistic interactions were expressed as the fractional inhibitory concentration index (FICI), which is calculated as the sum of MICs of the combination divided by the MICs of the active ingredients alone: FICI = MIC of active ingredient A in combination/MIC of active ingredient A alone + MIC of active ingredient B in combination/MIC of active ingredient B alone.

#### 2.2.7. Statistical Analysis

A multiple group comparison model with two independent factors using two-way ANOVA was used. Tukey’s multiple comparisons post-test was also utilized. All analyses were performed using GraphPad Prism (8.0.2) and Microsoft Excel 365 (2024) software.

## 3. Results and Discussion

### 3.1. Identification of the Chemical Components of Syzygium aromaticum Essential Oil

**Figure 3:**
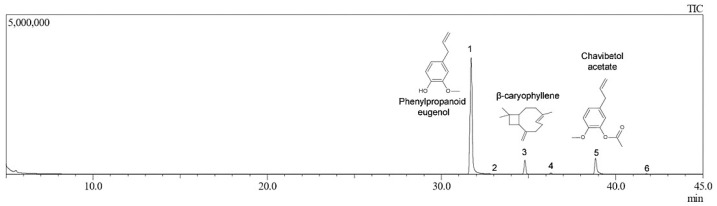
Chemical components of the *S. aromaticum* essential oil identified by GC-MS: Phenylpropanoid eugenol, β-caryophyllene, and Chavibetol acetate.

Chemical components of the *S. aromaticum* essential oil were identified by GC-MS, the chromatogram is shown in Figure 3. Table 5 contains the concentrations of compounds in clove oil that were identified by GC-MS and quantified by GC-FID. The major compound and the main secondary compounds were identified totaling 98.9% of the total compounds that constitute clove oil (Table 5). The main compound identified was the Phenylpropanoid eugenol, comprising 80.09%. This value aligns with the literature data, as the concentration of eugenol in clove oil can vary from 70 to 90% depending on geographic location, storage, and extraction methods, among other intrinsic and extrinsic factors affecting plant metabolism [13,28]. Other secondary compounds found in CO were β-caryophyllene and Chavibetol acetate with 7.74 and 11.07%, respectively (Table 5).

### 3.2. First Experimental Design and Selection of the Amphotericin B-Free Formulation

The first experimental design revealed that formulations with higher CO concentrations had a white color and a milky consistency, while those with lower CO concentrations were more translucent. All formulations exhibited a slightly acidic pH of 5.5. The increase in CO concentration in relation to the surfactant increased particle size and PDI, and this was adjudged as a bad result. The increase in surfactant concentration resulted in a reduction in particle size. For example, the formulation with 5% CO and 10% Pluronic^®^ F-127 (NESAF-06) had an average particle size of 28.5 nm (Table 2). When the CO concentration increased to 10% (NESAF-07), the particle size grew to 52 nm. These findings were consistent with those reported by Siqueira and co-researchers [13].

**Figure 4:**
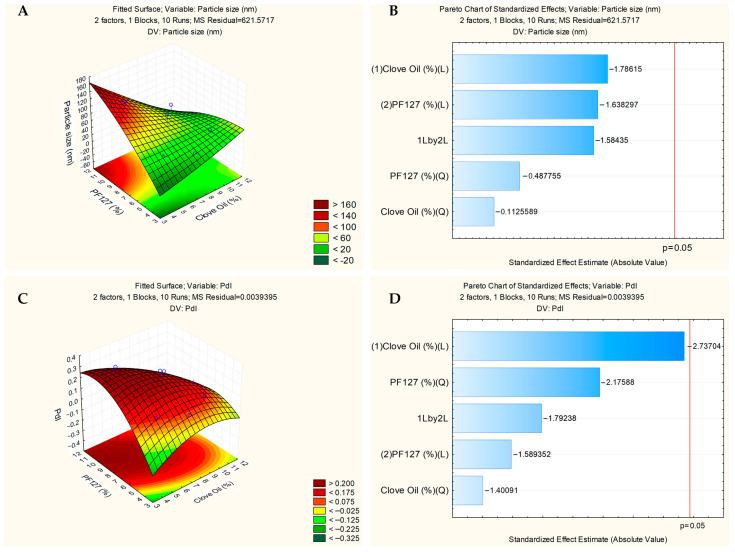
Initial experimental design of the nanoformulations without drug demonstrating the effects of concentration in relation to surfactant (Pluronic^®^ F127) and clove oil (CO) in the particle size: surface graph (**A**) and Pareto chart (**B**); and PDI: surface graph (**C**) and Pareto chart (**D**).

The experimental design results of the nanoformulations obtained by the Statistica 10 software are shown in Figure 4. Figure 4A,C show the surface graphs that relate the concentration of CO and surfactant with particle size and PDI, respectively. It can be concluded that the size and PDI increase proportionally to the increase in the concentration of CO. However, the Pareto diagrams (Figure 4B,D) show that none of the parameters, CO and surfactant, respectively, were critical for the preparation of nanoformulations. The choice of the ideal formulation was the one that presented the smallest particle size, PDI, and highest CO concentration, thus NESAF-06 and NESAF-09 were chosen to encapsulate the drug.

### 3.3. Second Experimental Design and Formulation Selection with Drug

The NESAF-07 with 10% CO and 10% Pluronic^®^ F-127 from the first experimental design exhibited a slightly large particle size (above 52 nm) and had unacceptable liquid consistency (Table 2). Therefore, NESAF-09 with 7.5% CO and 10% Pluronic^®^ F-127 was selected as a promising option for encapsulating AmB, as it had average size of 35 nm, transparent appearance and good liquid consistency. NESAF-06 and NESAF-09 were chosen for a second experimental design of nanoformulations containing amphotericin B.

The nanoformulations of the second experimental design yielded promising results (Table 4). The NEMLB-03 formulation, containing 10% CO and 0.025% AmB, exhibited an average particle size of 53.2 nm with a PDI of 0.176. However, NEMLB-05 with 7.5% CO and 0.035% AmB showed a smaller average particle size of 34.6 with a PDI of 0.16. In addition, NEMLB-06 formulation, containing 5% CO and 0.025% AmB, exhibited a particle size of 32.1 nm with a PDI of 0.218. Thus, the selected nanoformulations were NEMLB-5 and NEMLB-6 and controls without drug were NESAF-09 and NESAF-06, respectively.

**Figure 5:**
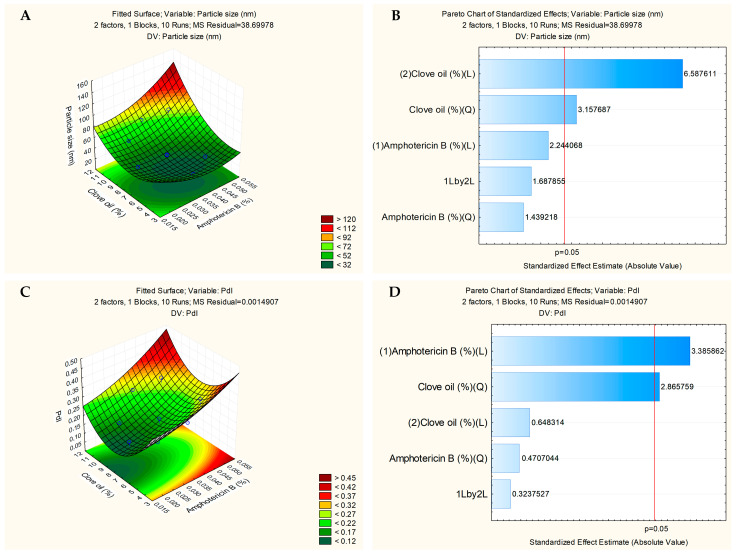
Experimental design of the nanoformulations with amphotericin B demonstrating the effects of concentration in relation to clove oil (CO) and amphotericin B in the size: surface graph (**A**) and Pareto chart (**B**); and PDI: surface graph (**C**) a Pareto chart (**D**).

Figure 5A shows the surface graph that relates the concentration of CO and amphotericin B with the particle size. Particle size increases with the addition of more CO to the formulation (Figure 5A). Analysis using Statistica 10 software demonstrated that CO significantly influenced particle size, and this result could be seen in the Pareto chart (Figure 5B). Furthermore, the increase in the amount of drug in the formulation was not critical and did not influence the particle size (Figure 5B).

Figure 5C shows the surface graph indicating the influence of CO and AmB on PDI. Both parameters influence the PDI. The PDI increases when CO and drug are added to the formulation. Furthermore, the increases in the amount of CO and drug were critical and influenced the PDI and this result could be seen in the Pareto chart (Figure 5D).

### 3.4. Physicochemical Characterization of the Nanoemulsions

**Figure 6:**
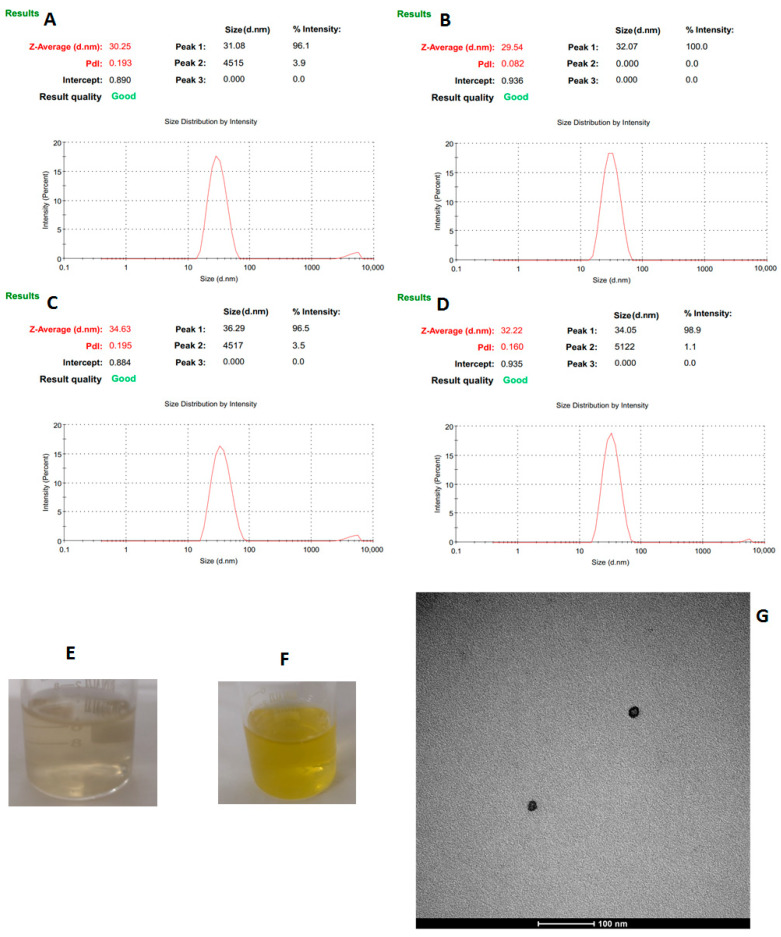
Average particle size (nm) of the nanoemulsions obtained by dynamic light scattering (DLS): NEMLB-06 (**A**), NESAF-06 (**B**), NEMLB-05 (**C**), NESAF-09 (**D**); NE without amphotericin B (**E**); NE containing amphotericin B (**F**) and transmission electron microscopy (TEM) image of the NE containing amphotericin B (NEMLB-05) (**G**).

NESAF-06 exhibited an average particle size of 29.5 nm and the AmB-containing formulation NEMLB-06 presented an average particle size of 30.2 nm (see Figure 6A,B). Both formulations maintained a pH of approximately 5.3. However, the formulations with higher concentrations of CO (7.5% instead of 5%) and AmB (0.035% instead of 0.025%) showed larger particle sizes; NESAF-09 measured 32.2 nm, while NEMLB-05 measured 34.6 nm (Figure 6C,D). These formulations also presented a slightly more acidic pH of 4.9, which is within a desirable range for topical applications in treating human skin infections [29].

In terms of visual appearance, the NEs without amphotericin B were colorless and translucent (Figure 6E). The formulations containing AmB appeared yellow, translucent with the characteristic scent of clove (Figure 6F). Transmission electron microscopy (TEM) images of the NE (NEMLB-05) revealed well-defined rounded shapes (Figure 6G).

The results confirmed the successful formulation of NEs with a satisfactory AmB content, excellent particle size (30–50 nm), and uniformity. The size and stability of the oil droplets depend on the type and concentration of oils and surfactants used. NEs effectively encapsulate lipophilic drugs, enhancing controlled drug release, physicochemical stability, and skin permeability, which prolongs therapeutic effects and allows lower doses, reducing side effects [30]. Earlier researchers developed NEs with castor oil, Transcutol^®^ P, Labrasol^®^, and Plurol^®^ Oleique containing AmB, with particle sizes of 112–126 nm and a PDI of 0.22, reinforcing the potential of NEs for AmB encapsulation [31].

### 3.5. Stability Studies

The formulations demonstrated good long-term stability. After 150 days, the formulation without AmB (NESAF-09) remained translucent when stored at 4 °C (Supplementary Material, Table S1), whereas the same formulation kept at 26 °C developed a slight yellowish tint. Similarly, the formulation containing AmB (NEMLB-05) exhibited a less intense yellow color under refrigeration compared to samples stored at room temperature, suggesting greater visual stability when kept refrigerated. Regarding particle size and PDI, no noticeable changes were observed during the stability studies (Supplementary Material, Table S2). This is consistent with a previous study where it was reported that NEs containing AmB maintained high stability under refrigeration for up to 365 days [32]. Furthermore, the NESAF-06 and NEMLB-06 formulations also remained stable during the stability study at 4 and 26 °C (Supplementary Material, Tables S1 and S2).

### 3.6. In Vitro Release Studies

**Figure 7:**
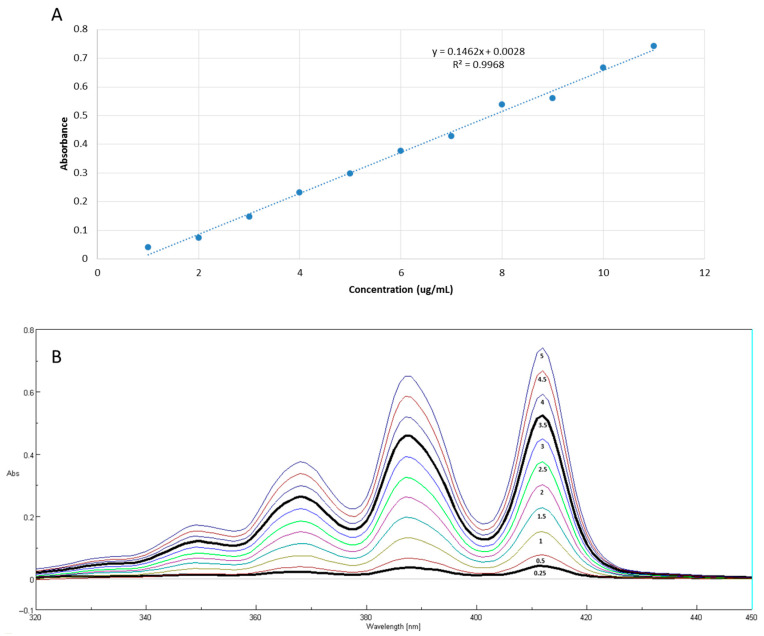
Analytical curve of amphotericin B obtained using a UV–VIS spectrophotometer at 412 nm (**A**); amphotericin B concentrations used to construct the analytical curve and absorption spectra (**B**).

The spectrophotometric method was validated before conducting the in vitro release studies. The maximum absorption of AmB in a phosphate buffer solution containing a solubilizing agent was at 412 nm, consistent with values reported in the literature [32]. Figure 7A shows the analytical curve of amphotericin B obtained using a UV–VIS spectrophotometer and Figure 7B shows amphotericin B concentrations used to construct the analytical curve with the absorption spectra.

The AmB content in NEMLB-05 was calculated using the same analytical curve, with results exceeding 93% of the content encapsulated in the nanoemulsion.

The AmB-free NEs showed absorption in a spectrophotometric range that does not interfere with AmB analysis. Therefore, AmB quantification (λ = 412 nm) in the presence of NEs was unaffected. Spectrophotometric analysis of AmB dilutions in the receptor medium with surfactant yielded an analytical curve with an R^2^ value greater than 0.99, confirming the method’s linearity (Figure 7A). This allowed for the percentage of AmB released from the NEs over time (hours) to be calculated.

**Figure 8:**
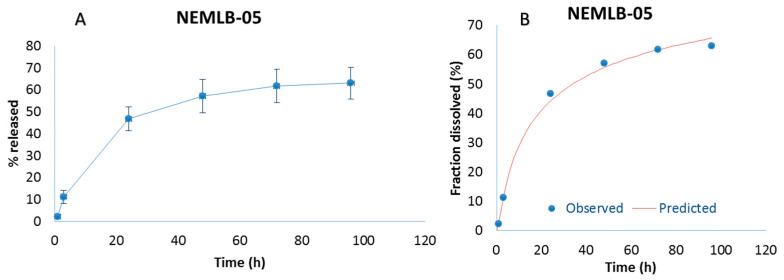
In vitro release profile of amphotericin B from the nanoemulsion (NEMLB-05) (**A**); graph obtained after fitting the release data with the Gompertz kinetic model using the DDSolver software (**B**).

Drug release (the amount of AmB in the NE that crossed the membrane and remained in the receptor medium) was higher in the first 24 h, followed by slow and sustained release over 96 h. A total of 62.95 ± 7.19% of AmB was released in 96 h with no burst effect for NEMLB-05 (Figure 8A). The NEs act as nanocarriers capable of transporting the drug in biological media with slow and sustained release.

A release study was also conducted using free AmB in DMSO. It resulted in minimal membrane crossing and very slow release in the receptor medium, with only 20 ± 0.82% of the active substance released over 96 h (Supplementary Material, Figure S1). The enhanced ability of NEs to sustain AmB release over a longer period is consistent with previous study, an indication that these carriers have the potential to improve AmB permeation and penetration through the skin [33].

Among the kinetic models evaluated (Supplementary Material, Table S3), the Gompertz model yielded the highest adjusted coefficient of determination (R^2^ = 0.9939) and MSC value (4.4314) (Figure 8B). This model can be applied to release profiles and is represented by the equation: F = 100 × *e*^−α×*e*[−β×log(t)]^, where F is the percentage dissolved at time t; α represents the proportion of undissolved substance at t = 1, described as the location or scale parameter; and β is the dissolution rate per unit of time, described as the shape parameter [22,34]. This model indicates a rapid release of the drug, with a sharp increase initially, followed by a gradual approach to an asymptotic maximum dissolution [22,34,35].

### 3.7. In Vitro Biological Activity of Clove Oil Nanoemulsions

**Figure 9:**
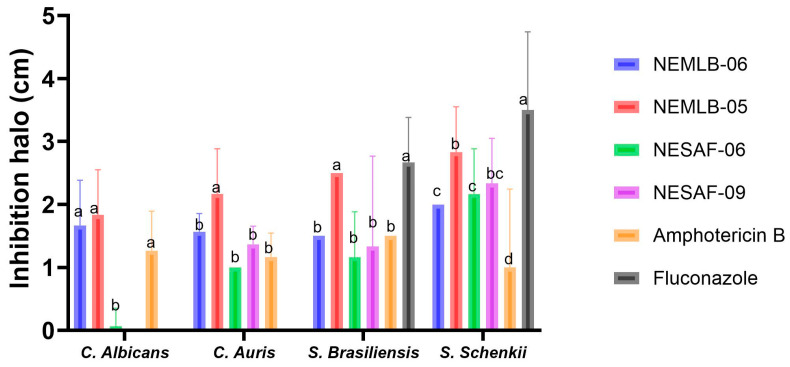
Fungal growth inhibition in the agar diffusion test. NEMLB-05 = 0.035% of drug and 7.5% CO, NEMLB-06 = 0.025% of drug and 5% CO, NESAF-06 = 5% of CO, NESAF-09 = 7.5% CO, AmB 0.035% (drug solution) and Fluconazol = 2.5 mg/mL (standard antifungal). Bar graph with confidence interval (95%) of the means of the inhibition zones of the tested formulations, by fungal strain. The letters indicate significant differences between the formulations for each strain (*p* < 0.05; Tukey test).

In the antifungal test using agar diffusion, the AmB-containing NE (NEMLB-05) effectively inhibited *C. auris*, with the largest inhibition zone (2.17 cm), compared to *C. albicans* (1.83 cm). Furthermore, in *C. auris*, the NEMLB-05 formulation presented the greatest inhibition zone with a significant difference in relation to the other formulations and the control amphotericin B (Figure 9).

In *Candida albicans*, the formulations NEMLB-06, NEMLB-05, and amphotericin B presented the highest inhibition zones and were statistically grouped with the letter “a”, indicating that they did not present significant differences between them. Halos showing growth inhibition of *C. albicans* and *C. auris* can be seen on culture medium plates (Supplementary Material, Figure S3). Fluconazole is a standard antifungal used to treat mycoses susceptible to this drug. Fluconazole was not able to inhibit the growth of *C. albicans* and *C. auris*, as the fungi were probably resistant to the antifungal agent used in the experiment. *C. albicans* and emerging *Candida* ssp. may develop resistance to fluconazole due to mutations in genes involved in ergosterol synthesis and increased expression of efflux pumps [36].

NEMLB-05 formulation effectively inhibited *S. brasiliensis* and *S. schenkii*. The formulation NEMLB-05 and fluconazole presented the largest inhibition halos for *S. brasiliensis*, with a significant difference in relation to the other formulations and the control amphotericin B. Fluconazole presented the largest inhibition zone in *S. schenckii*. NEMLB-05 and NESAF-09 presented similar inhibition zones in this strain. NESAF-06 presented a significant difference with NEMLB-05, but no statistical difference with NESAF-09. Lastly, NEMLB-06 had inhibition equivalent to NESAF-06, while amphotericin B presented the smallest inhibition zone in *S. schenckii* (Figure 9).

Fungal growth inhibition in the agar diffusion test was important for selecting nanoformulations for the next study of antimicrobial activity in the 96-well plate assay. Nanoformulations were selected based on the best antifungal activity: NEMLB-05 and NESAF-09, using free amphotericin B as the control.

The in vitro results of the antifungal activity are showed in the Table 6. In the treatment with NEMLB-05 containing amphotericin B, no statistically significant differences in IC_50_ were observed between the fungal strains. For free amphotericin B, *C. auris* did not present IC_50_ values (values > 0.25 mg/mL), while in the other three strains, no statistical difference was presented. NESAF-09 showed a higher IC_50_ in *C. auris* when compared to the other three strains with a statistically significant difference. The lowest IC_50_ concentrations were observed for *S. schenckii* and *C. albicans*, and no statistically significant difference was observed. NESAF-09 acting on *S. schenckii* shows lower IC_50_ than *S. brasiliensis* with a statistically significant difference. In the group treated with NEMLB-05 in relation to clove oil, *C. albicans* showed a significant difference in the IC_50_ in relation to *C. auris*, *S. schenckii*, and *S. brasiliensis*. The IC_50_ of *C. auris*, *S. schenckii*, and *S. brasiliensis* are similar and there is no statistically significant difference. There is a statistically significant difference between the IC_50_ of NEMLB-05 in relation to free amphotericin B for all strains evaluated. In the IC_50_ of NEMLB05 in relation to NESAF-09, there is a statistically significant difference in relation to *C. auris*, *C. albicans*, and *S. brasiliensis*, but there is no difference in relation to *S. schenkii*.

There was a synergistic antifungal effect between amphotericin B and CO, which was observed by the reduction in the IC_50_ values of the drug and CO. The IC_50_ values in *C. albicans* were compared and NEMLB-05 demonstrated antifungal activity that was 3.6 times greater than that of the free amphotericin B and 3.8 times greater than that of the NESAF-09 (Table 6).

In relation to *C. auris*, NEMLB-05 demonstrated excellent antifungal activity with IC_50_ of 0.0117 mg/mL (2.3801 mg/mL in relation to CO) which was 21 times higher than that of free amphotericin B (0.25 mg/mL) and 3.2 times greater than that of the NESAF-09 (7.7016 mg/mL of CO). NEMLB-05 showed promising results against *C. auris*, as free AmB was unable to inhibit its growth (Table 6). *Candida auris* is known for its thermal tolerance to high temperatures (~42 °C), unique cell wall characteristics, and strong adaptability, contributing to its resistance to current antifungals [4,5]. Given that *C. auris* poses a growing public health threat due to resistance to existing antifungals, NEMLB-05 demonstrates promising potential against *C. auris*. [5,9]. The nanoformulation without amphotericin B (NESAF-09) also showed satisfactory antifungal activity against *C. albicans* and *C. auris*, as it contains CO that presents antifungal activity against *Candida* strains [13]. Furthermore, there was a synergistic antifungal effect of the CO in relation to amphotericin B, as NEMLB-05 is 3.8 and 3.2 times more potent against *C. albicans* and *C. auris*, respectively, than NESAF-09 in relation to the concentration of CO (Table 6).

The nanoformulation NEMLB-05 showed excellent antifungal activity with IC_50_ of 0.0165 mg/mL and 0.0090 mg/mL for *S. brasiliensis* and *S. schenkii*, respectively. The nanoformulation without amphotericin B (NESAF-09) also showed satisfactory antifungal activity against *Sporothrix* strains (Table 6). In some regions of Brazil, such as Rio de Janeiro, cases of Sporotrichosis in cats are increasing [37,38]. This infection can be transmitted to humans through scratches or bites, as cats are kept as pets, which can facilitate transmission. The treatment of Sporotrichosis is long and difficult and there are cases of resistance of the *S. brasiliensis* to antifungals such as fluconazole and itraconazole. Thus, nanoformulations are a topical therapeutic alternative for the treatment of skin lesions caused by *Sporothrix* ssp.

There is a synergistic antifungal effect between amphotericin B and CO demonstrated in the results of Table 6. The nanoformulation (NESAF-09) is composed of CO with antifungal action and the target is the cell membrane, as well as amphotericin B with proven action on the fungal membrane. Regarding the mechanism of action, AmB binds to ergosterol in the cell membrane, causing pore formation, cytosolic leakage, and cell death. One of the potential mechanisms behind the antifungal activity of eugenol, the main component of CO, is related to the importance of its phenolic group, which has hydrogen-binding capacity, electron delocalization, and acidity [39]. These properties contribute to the disruption of yeast cell membranes, altering their fluidity and permeability, and leading to cytoplasmic leakage [40]. The mechanism of action of CO on the fungal membrane has already been observed by electron microscopy, as it causes morphological changes and cell deformation in yeasts treated with essential oils. The deformations and changes in the fluidity of the fungal membrane caused by essential oils can increase the permeability to drugs and the sensitivity of the microorganism to antifungals. This synergy led to a greater inhibition of fungal growth, thereby reducing the amount of medication required to exert its antifungal effects [41,42].

**Figure 10:**
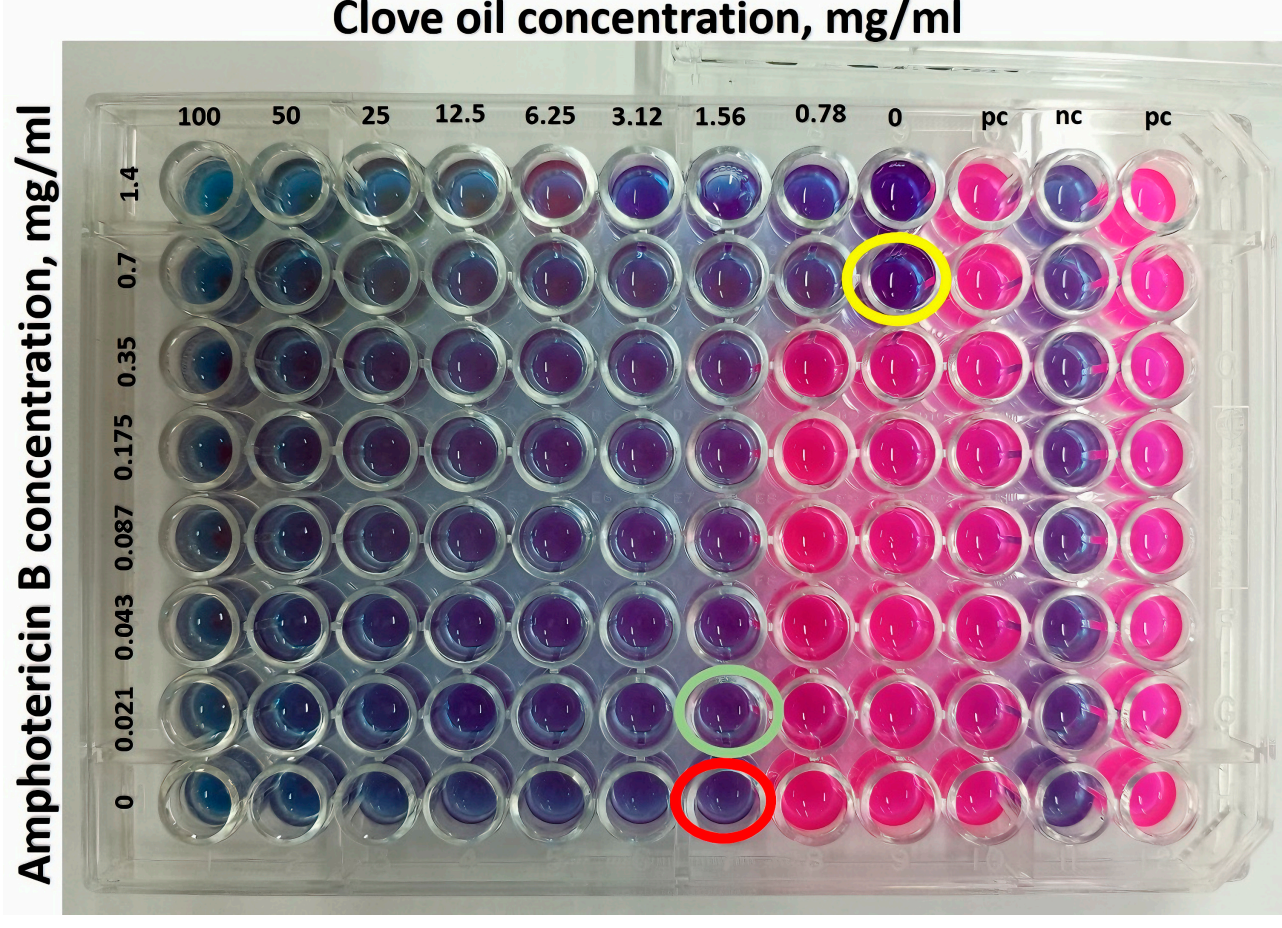
Evaluation of a checkerboard assay in the 96-well microtiter plate. The two rightmost columns contain the positive growth control (pc) and negative growth control (nc) to confirm bacterial growth without CO, AMB, and medium sterility. The red circle shows the clove oil MIC and yellow circle shows the AmB MIC. The green circle indicates the combination that resulted in a synergistic effect on the C. auris strain, for which the FICI calculation should be (0.021875/1.5625) + (0.7/1.5625) = 0.462.

The checkerboard assay demonstrated that there was synergism between clove oil and AmB in relation to the inhibition of the growth of the fungus *C. auris*, as can be seen in Figure 10. The FICI showed a value of 0.462 (<0.5). This confirms the results obtained in the other biological tests presented in this article.

The essential oils can be used to treat fungal diseases. Earlier researchers observed the antifungal effect of essential oils on *C. albicans*, as it reduced the growth of the fungus [43]. Another group of researchers demonstrated the antifungal activity of essential oils on *C. auris*; the essential oils with the greatest antifungal activity were lemongrass, clove, and cinnamon [44]. Furthermore, other researchers developed a nanoformulation containing CO and their results demonstrated that the nanoformulation exhibited greater antifungal activity than free clove oil against *C. albicans* and *C. glabrata*. Nanotechnology has promoted the antimicrobial activity of essential oils because the nanometric size of nanoemulsions can cause greater interaction between the active ingredients of essential oils and pathogenic microorganism [13,45].

A likely mechanism of action of the NEMLB-05 containing CO and amphotericin B is its ability to aim for two or more biological targets simultaneously. The possible mechanisms of action include the degradation or fragmentation of nuclear DNA and the cell wall, as well as increased mitochondrial hyperpolarization and increased cytoplasmic calcium concentrations. Thus, this synergy is a promising therapeutic alternative for the treatment of skin lesions caused by fungal diseases [46,47].

Ambisome^®^ (liposomal medication) and Abelcet^®^ (lipid complex) are formulations containing AmB designed to improve the therapeutic index. Although these formulations have fewer side effects, they still have cases of nephrotoxicity and high costs, raising concerns about their use in the treatment of fungal infections [48,49,50,51]. Several research groups have developed new AmB formulations using nanotechnology to promote the topical treatment of fungal diseases. A systemic fungal infection can occur when treatment is inadequate, especially when the fungus initially affects the skin. Therefore, formulations that effectively release the drug to the fungus, while it is still confined to the skin are of interest, as they would avoid the need for systemic treatment with AmB. A liposome developed by Perez et al. (2016) showed antifungal activity, with a higher concentration of AmB accumulated in the stratum corneum and epidermis compared to Ambisome^®^ [52].

Pharmaceutical nanotechnology has contributed significantly to the development of more effective therapies using nanoemulsions [53]. Thus, nanoformulations based on CO nanoemulsions (NESAF-09) and nanoemulsions containing amphotericin B (NEMLB-05) are a promising alternative for the treatment of cutaneous lesions caused by *Sporothrix* spp. and *Candida* spp. in animals and humans. Furthermore, nanoemulsions can also be associated with Ambisome^®^ or Abelcet^®^, systemic treatment, with a greater possibility of cure.

## 4. Conclusions

Nanoemulsions based in clove oil (CO) containing amphotericin B were successfully produced. The composition of the nanoformulation was optimized by experimental design and it shows that the average particle size is influenced by the concentrations of CO and surfactant with less influence of the drug. The more clove oil, the larger the particle size and PDI. However, the larger the amount of surfactant, the smaller the particle size, up to a limit of concentration used. The study also revealed the significant impact of CO concentration on the average particle size of the NEs. NEMLB-05 demonstrated long-term stability (150 days) under refrigeration (4 °C) and sustained release, outperforming free AmB. The nanoemulsion containing amphotericin B, proposed for topical application in this study, showed promising antifungal activity against *C. albicans*, *C. auris*, *S. brasiliensis*, and *S. schenkii*. Nanotechnology has promoted the antimicrobial activity of drugs and essential oils because the nanometric size of nanoemulsions can cause greater interaction between the active compounds and the microorganism. Additionally, increased antimicrobial activity was observed when AmB was encapsulated in nanoemulsion, compared to free drug against *C. auris*. Developing new treatments for this fungus, especially in cases of resistance, is important for public health. Given the potential of these NEs, additional studies using in vivo models and formulation improvements are needed to advance clinical studies.

## Figures and Tables

**Table 1:** First CCD experimental design with a central point.

Independent Factors	(wt/vol) (%)	Level	(wt/vol) (%)	Level	(wt/vol) (%)	Level
Clove oil	5	Low (−1)	7.5	Center (0)	10	High (1)
Pluronic^®^ F-127	5	Low (−1)	7.5	Center (0)	10	High (1)

Nanoformulation volume = 10 mL.

**Table 2:** Formulations produced in the first experimental design.

Formulation	Clove Oil (wt/vol) (%)	Pluronic^®^ F-127 (wt/vol) (%)	Particle Size (nm)	PDI
NESAF-01	5	5	35.170 ± 3.352	0.120 ± 0.045
NESAF-02	10	5	114.800 ± 1.697	0.314 ± 0.011
NESAF-03 *	7.5	7.5	50.030 ± 3.762	0.187 ± 0.041
NESAF-04	11	7.5	88.633 ± 12.258	0.271 ± 0.077
NESAF-05	4	7.5	29.825 ± 2.326	0.108 ± 0.095
NESAF-06	5	10	28.523 ± 0.675	0.072 ± 0.012
NESAF-07	10	10	52.860 ± 0.509	0.122 ± 0.028
NESAF-08	7.5	4.0	90.423 ± 5.597	0.318 ± 0.030
NESAF-09	7.5	11	35.735 ± 8.436	0.117 ± 0.066

Nanoformulation volume = 10 mL. * Central point.

**Table 3:** Second CCD experimental design with a central point.

Independent Factors	(wt/vol) (%)	Level	(wt/vol) (%)	Level	(wt/vol) (%)	Level
Amphotericin B	0.025	Low (−1)	0.035	Center (0)	0.045	High (1)
Clove oil	5	Low (−1)	7.5	Center (0)	10	High (1)

Nanoformulation volume = 10 mL.

**Table 4:** Formulations produced in the second experimental design: nanoformulation with amphotericin B.

Formulation	Clove Oil (wt/vol) (%)	Amphotericin B (wt/vol) (%)	Particle Size (nm)	PDI
NEMLB-01	10	0.045	75.870 ± 6.817	0.328 ± 0.025
NEMLB-02	7.5	0.049	41.070 ± 3.677	0.242 ± 0.032
NEMLB-03	10	0.025	53.265 ± 1.916	0.176 ± 0.023
NEMLB-04	4	0.035	32.055 ± 0.587	0.247 ± 0.045
NEMLB-05 *	7.5	0.035	34.615 ± 0.587	0.160 ± 0.006
NEMLB-06	5	0.025	32.155 ± 0.049	0.218 ± 0.028
NEMLB-07	5	0.045	38.810 ± 2.036	0.397 ± 0.105
NEMLB-08	7.5	0.021	38.080 ± 1.273	0.161 ± 0.012
NEMLB-09	11	0.035	67.215 ± 1.421	0.258 ± 0.011

Nanoformulation volume = 10 mL. * Central point.

**Table 5:** Chemical components of the *S. aromaticum* essential oil identified by GC-MS and quantified by GC-FID.

N.	RT	AI_rep_	AI_calc_	Substances	Relative Abundance (%)
1	31.706	1362	1356	Phenylpropanoid eugenol	80.09
2	32.726	1384	-	n.i.	0.12
3	34.788	1431	1427	β-caryophyllene	7.74
4	36.279	1466	-	n.i.	0.72
5	38.834	1526	1524	Chavibetol acetate	11.07
6	41.773	1599	-	n.i.	0.26
Total Identified					98.90

n.i. = not identified.

**Table 6:** Antifungal activity of nanoformulations and free drug.

	IC_50_ (mg/mL)		
Fungal Strains	NEMLB-05 (mg/mL of Drug)	Free Amphotericin B (mg/mL of Drug)	NESAF-09 (mg/mL of CO)
	(mg/mL of CO)		
*Sporothrix brasiliensis* ATCC 5110 (S5)	AmB: 0.0165 ± 0.0160 ^d^	0.1464 ± 0.0925 ^d^	
	CO: 2.1900 ± 1.241 ^c,e^		3.9929 ± 1.0829 ^a,b,e^
*Sporothrix schenkii* ATCC 15,383 (S15)	AmB: 0.0090 ± 0.0004 ^d^	0.1253 ± 0.0504 ^d^	
	CO: 1.9191 ± 0.0842 ^c^		2.5102 ± 0.9016 ^a,b^
*Candida albicans* ATCC SC5314	AmB: 0.0207 ± 0.0160 ^d^	0.0744 ± 0.0630 ^d^	
	CO: 0.7667 ± 0.3717 ^c,e^		2.9110 ± 0.2705 ^a,e^
*Candida auris* ATCC MYA-5002	AmB: 0.0117 ± 0.0016 ^d^	>0.25 ^d^	
	CO: 2.3801 ± 0.1386 ^c,e^		7.7016 ± 1.0455 ^a, e^

NEMLB-05 = 0.035% of amphotericin B and 7.5% of CO; NESAF-09 = 7.5% of CO without drug; CO = clove oil; free AmB = 0.025%. ^a,b,c^ statistically significant differences in relation to formulation (column) (*p* < 0.05; Tukey test). ^d,e^ statistically significant differences in relation to NEMLB-05 and free AmB (line) (*p* < 0.05; Tukey test).

## Data Availability

Additional data may be available upon request via email.

## Supplementary Materials

The following supporting information can be downloaded at https://www.mdpi.com/article/10.3390/pharmaceutics17070925/s1, Table S1. Stability study: Average particle size and polydispersity index (PDI) of the nanoemulsions without AmB; Table S2. Stability study: Average particle size and polydispersity index (PDI) of the nanoemulsions with AmB; Table S3. Kinetic models evaluated in the release study. Adjusted determination coefficients (R^2^) and MSC (model selection criterion) obtained after adjusting the kinetic models using the DDSolver software; Figure S1. In vitro release profile of free amphotericin B; Figure S2. Inhibition of Candida auris (A) and Candida albicans (B): NEMLB-06 (1), NEMLB-05 (2), NESAF-06 (3), NESAF-09 (4), free amphotericin B (5), water (6), and fluconazole (7).
