# Supplementary material for: Clove Oil-Based Nanoemulsion Containing Amphotericin B as a Therapeutic Approach to Combat Fungal Infections
Source: Pharmaceutics. 2025 Jul 17;17(7):925. doi: 10.3390/pharmaceutics17070925 (PMC12300589; doi:10.3390/pharmaceutics17070925)
Supplement: Supplementary file 1 [file pharmaceutics-17-00925-s001.zip › pharmaceutics-3735197-supplementary.pdf]

Supplementary material

Table S1. Stability study: Average particle size and polydispersity index (PDI) of the nanoemulsions without AmB.

Condition	Storage days	Particle size (nm)		PDI	
**Room temperature**		NESAF-06	NESAF-09	NESAF-06	NESAF-09
**(26ºC)**	00	28.52±0.67	30.88±1.89	0.07±0.01	0.12±0.050
	08	29.52±0.52	31.83±0.76	0.13±0.05	0.07±0.005
	45	29.68±1.89	30.79±0.56	0.21±0.07	0.09±0.040
	80	26.63±0.54	28.85±0.40	0.13±0.04	0.13±0.024
	115	29.41±1.94	29.36±0.52	0.17±0.03	0.07±0.015
	150	27.46±0.89	29.37±0.50	0.14±0.06	0.07±0.010
**Under refrigeration**					
**(4ºC)**	08	30.61±1.16	31.89±0.86	0.13±0.07	0.09±0.019
	45	30.21±1.01	31.24±0.94	0.19±0.04	0.09±0.027
	80	28.79±0.06	30.60±0.70	0.19±0.06	0.11±0.009
	115	30.28±1.48	30.64±0.32	0.16±0.05	0.13±0.114
	150	29.96±1.44	31.83±0.39	0.17±0.07	0.20±0.032

Table S2. Stability study: Average particle size and polydispersity index (PDI) of the nanoemulsions with AmB.

Condition	Storage days	Particle size (nm)		PDI	
**Room temperature (26ºC)**		NEMLB-06	NEMLB-05	NEMLB-06	NEMLB-05
	01	32.15±0.05	34.61±0.59	0.22±0.030	0.16±0.010
	07	32.14±0.80	34.64±1.12	0.21±0.034	0.21±0.031
	18	31.99±1.29	34.42±0.08	0.21±0.047	0.22±0.004
	53	31.48±0.93	33.58±0.68	0.25±0.037	0.23±0.033
	100	29.00±021	32.12±0.21	0.14±0.033	0.18±0.013
	150	29.66±1.85	31.07±0.51	0.20±0.060	0.17±0.014
**Under refrigeration**					
**(4ºC)**	07	33.43±1.10	37.190±0.66	0.24±0.031	0.24±0.032
	18	32.27±0.33	34.903±0.32	0.21±0.034	0.23±0.016
	53	34.15±1.51	35.593±1.17	0.26±0.028	0.24±0.025
	100	32.11±1.84	34.683±0.46	0.22±0.066	0.22±0.017
	150	33.85±1.16	36.050±0.92	0.27±0.060	0.22±0.038

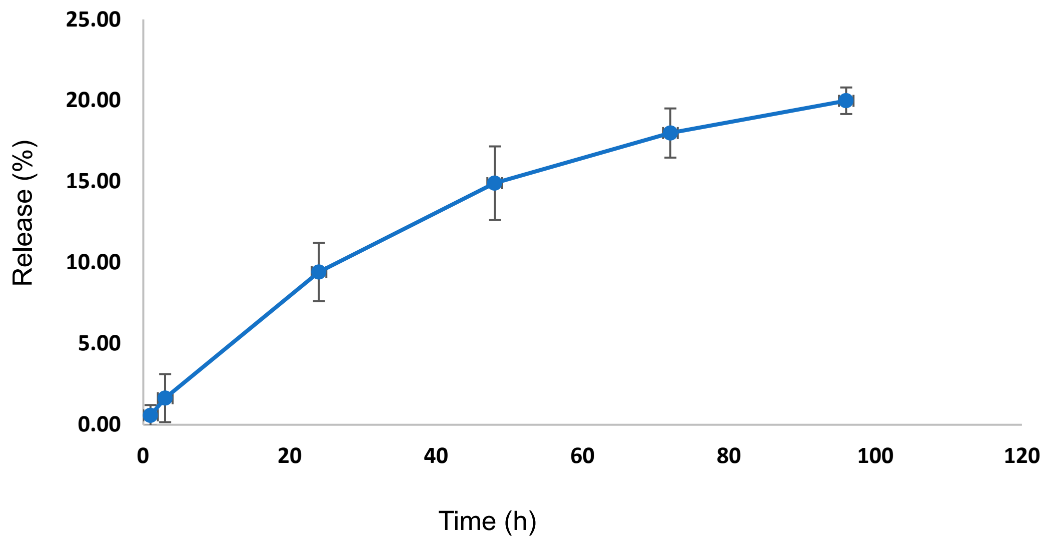

Figure S1. *In vitro* release profile of free amphotericin B.

Table S3. Kinetic models evaluated in the release study. Adjusted determination coefficients (R^2) and MSC (Model Selection Criterion) obtained after adjusting the kinetic models using the DD-solver software.

MODEL	R^2-Adj NEMLB-05	MSC NEMLB-05
Zero Order	0.618	0.629
First order	0.817	1.366
Higuchi	0.927	2.285
Korsmeyer-Peppas	0.831	1.334
Hixon-Crowell	0.765	1.116
Hopfenburg	0.707	0.783
Baker-Lonsdale	0.932	2.355
Peppas-Salin	0.917	1.999
Welbull	0.896	1.776
Logistic	0.955	2.663
**Gompertz***	**0.995***	**4.810***
Probit	0.973	3.176

R^2_Adj= coefficient of determination adjusted according to the DD-solver software, MSC = Model Selection Criteria.

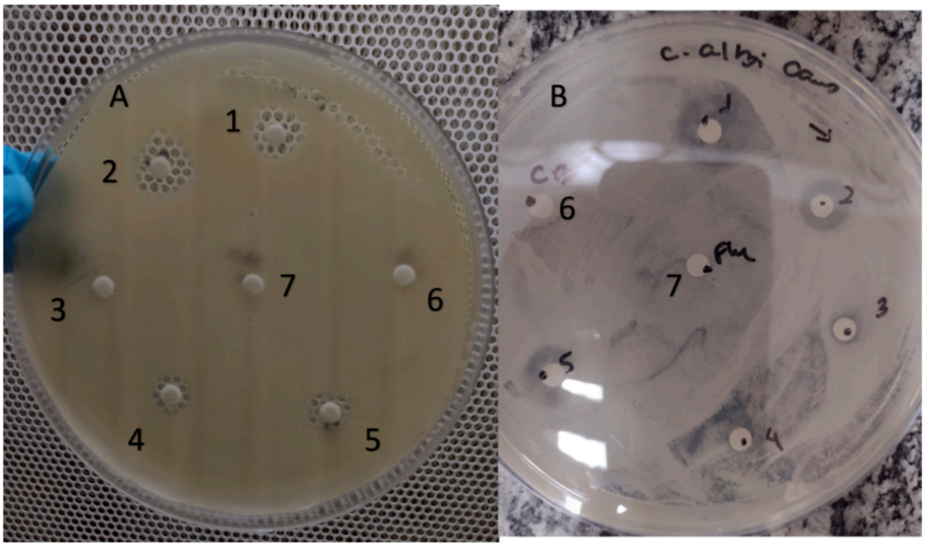

Figure S2. Inhibition of *Candida auris* (A) and *Candida albicans* (B): NEMLB-06 (1), NEMLB-05 (2), NESAF-06 (3), NESAF-09 (4), free amphotericin B (5), water (6) and fluconazole (7).
